# Effect of Magnetic Resonance Image Quality on Structural and Functional Brain Connectivity: The Maastricht Study

**DOI:** 10.3390/brainsci14010062

**Published:** 2024-01-08

**Authors:** Joost J. A. de Jong, Jacobus F. A. Jansen, Laura W. M. Vergoossen, Miranda T. Schram, Coen D. A. Stehouwer, Joachim E. Wildberger, David E. J. Linden, Walter H. Backes

**Affiliations:** 1 Department of Radiology and Nuclear Medicine, Maastricht University Medical Centre, 6202 AZ Maastricht, The Netherlands; 2 School for Mental Health and Neurosciences (MHeNs), Maastricht University, 6200 MD Maastricht, The Netherlands; 3 Department of Internal Medicine, Maastricht University Medical Centre, 6202 AZ Maastricht, The Netherlands; 4 School for Cardiovascular Disease (CARIM), Maastricht University, 6200 MD Maastricht, The Netherlands; 5 Heart and Vascular Centre, Maastricht University Medical Centre, 6202 AZ Maastricht, The Netherlands

**Keywords:** brain connectivity, image quality, magnetic resonance imaging, population-cohort study, neuroimaging

## Abstract

In population-based cohort studies, magnetic resonance imaging (MRI) is vital for examining brain structure and function. Advanced MRI techniques, such as diffusion-weighted MRI (dMRI) and resting-state functional MRI (rs-fMRI), provide insights into brain connectivity. However, biases in MRI data acquisition and processing can impact brain connectivity measures and their associations with demographic and clinical variables. This study, conducted with 5110 participants from The Maastricht Study, explored the relationship between brain connectivity and various image quality metrics (e.g., signal-to-noise ratio, head motion, and atlas–template mismatches) that were obtained from dMRI and rs-fMRI scans. Results revealed that in particular increased head motion (R^2^ up to 0.169, *p* < 0.001) and reduced signal-to-noise ratio (R^2^ up to 0.013, *p* < 0.001) negatively impacted structural and functional brain connectivity, respectively. These image quality metrics significantly affected associations of overall brain connectivity with age (up to −59%), sex (up to −25%), and body mass index (BMI) (up to +14%). Associations with diabetes status, educational level, history of cardiovascular disease, and white matter hyperintensities were generally less affected. This emphasizes the potential confounding effects of image quality in large population-based neuroimaging studies on brain connectivity and underscores the importance of accounting for it.

## 1. Introduction

Population-based cohort studies are extremely relevant sources of fundamental research data, contribute to a better understanding of health effects of life styles and pathophysiology of diseases, and reveal key information on risk factors [1]. If the structure and function of the brain are of interest, neuroimaging using magnetic resonance imaging (MRI) is often the preferred tool to incorporate into the study design. Neuroimaging can provide valuable structural and functional information on the brain, but the large amount of individuals in combination with the typical size of MRI data poses certain challenges in terms of data acquisition, storage, processing, and analysis [2]. Although there are several on-going large-scale neuroimaging population-based cohort studies, e.g., the Generation R Study [3], Rotterdam Scan Study [4], UK Biobank [5], Human Connectome Project [6,7], The Rhineland Study [8], and The Maastricht Study [9], each using different scanner hardware, study-specific scan protocols, and processing tools, there is no consensus on data acquisition and processing. In order to recognize potential biases introduced during data acquisition and processing, it is important to be transparent about the quality of the MRI data itself and the way these data are processed. 

In the last two decades, advanced MRI techniques have been developed that allow mapping of the connectivity of the brain’s network. Two main techniques to do so are typically diffusion-weighted MRI (dMRI) and resting-state functional MRI (rs-fMRI). The dMRI estimates the axonal orientations which are consecutively used to calculate white matter fiber tracts between brain regions, i.e., structural connectivity, using tractography algorithms [10,11]. The rs-fMRI data are used to calculate functional connectivity as the correlation between temporal changes in the blood-oxygen-level-dependent (BOLD) signal of spatially distinct brain regions [12,13]. 

Recent research has indicated that not only in neurological and psychiatric disorders [14], but also in systemic conditions such as type 2 diabetes mellitus (T2DM), structural [15,16,17] as well as functional brain connectivity [18,19,20,21] are altered compared to healthy controls. 

Previous research has shown that head motion is an important confounder for measures derived from structural MRI scans, even when visible artifacts are removed [22,23], as well as for brain connectivity measures obtained from dMRI and rs-fMRI scans [24,25,26]. Other sources of image-quality-induced bias include limitations in the signal-to-noise ratio (SNR) [27,28] and magnetic field inhomogeneities leading to geometric distortions, which in turn can result in misalignment (spatial mismatch) between dMRI and rs-fMRI data and brain atlases [29,30]. 

These studies, however, have a more narrowed scope. Either the number of participants in these studies is relatively small compared to the typical number of participants in population imaging studies, which often have thousands of participants, or the study population was limited to children and younger adults. Also, to the best of our knowledge, it has not been shown how strong image quality influences the associations between network measures and demographic and clinical variables, which is of particular interest to evaluate and eventually compare ongoing population imaging studies as being relevant in the clinical and healthcare context. Lastly, the aforementioned studies focused on either dMRI or rs-fMRI, while the results may be extended from rs-fMRI to dMRI or vice versa.

We implemented a quality assessment procedure within the structural and functional brain connectivity processing pipeline of The Maastricht Study [9], which focuses on the etiology, pathophysiology, complications, and comorbidities of T2DM and in which both dMRI as well as rs-fMRI data were acquired in more than 5000 participants with ages between 40 and 75 years. This gives us the opportunity to determine how the effect of image quality is expressed in a more older and larger population than reported from previous studies with smaller sample sizes and/or younger cohorts. Therefore, the main aim of the current study was to investigate how strong structural and functional connectivity outcome measures are related to image quality metrics of SNR, head motion, and atlas mismatch. Secondly, we were interested to what extent image quality affects associations between brain connectivity and typical demographic and clinical variables of interest, i.e., age, sex, body mass index, diabetes status, educational level, history of cardiovascular disease, and white matter hyperintensities. Third, we studied which of these demographic and clinical variables were most strongly associated with low image quality. 

For clarity and where applicable per section, we first report the methods and results for dMRI/structural connectivity followed by those for rs-fMRI/functional connectivity.

## 2. Materials and Methods

### 2.1. Study Population

**Figure A1:**
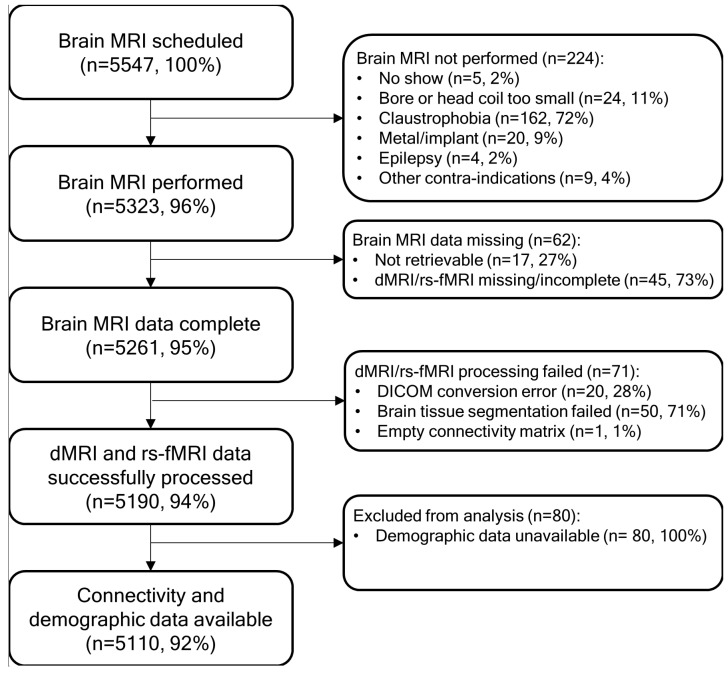
Flowchart showing the number of participants for whom both structural and functional brain connectivity measures could be calculated successfully and demographic data were available.

We used data from The Maastricht Study, an observational population-based cohort study. The rationale and methodology have previously been described [9]. In brief, the study focuses on the etiology, pathophysiology, complications, and comorbidities of T2DM and is characterized by an extensive phenotyping approach. Eligible for participation were all individuals aged between 40 and 75 years and living in the southern part of the Netherlands. Participants were recruited through mass media campaigns, the municipal registries, and the regional Diabetes Patient Registry via mailings. Recruitment was stratified according to known type 2 diabetes status, with an oversampling of individuals with T2DM for reasons of efficiency. Structural, diffusion, and resting-state functional MRI measurements were implemented from December 2013 onward to February 2017 and were completely available in 5261 (95%) of 5547 participants. Processing of the dMRI or rs-fMRI data failed in 71 participants, and in the remaining 5190 participants (94%) complete data on covariates were available in 5110 (92%, a flow chart is provided in Figure A1). The study has been approved by the institutional medical ethics committee (NL31329.068.10) and the Minister of Health, Welfare and Sports of the Netherlands (permit 131088-105234-PG). All participants gave written informed consent.

### 2.2. MRI Data Acquisition and Retrieval

For each participant, MRI data were acquired on a 3T clinical magnetic resonance scanner (MAGNETOM Prisma^fit^, Siemens Healthineers GmbH, Munich, Germany) located at a dedicated scanning facility (Scannexus, Maastricht, The Netherlands) using a head/neck coil with 64 elements for parallel imaging. The MRI protocol included a three-dimensional (3D) T1-weighted (T1w) magnetization prepared rapid acquisition gradient echo (MPRAGE) sequence (repetition time/inversion time/echo time (TR/TI/TE) 2300/900/2.98 ms, 176 slices, 256 × 240 matrix size, 1.0 mm cubic reconstructed voxel size); a fluid-attenuated inversion recovery (FLAIR) sequence (TR/TI/TE 5000/1800/394 ms, 176 slices, 512 × 512 matrix size, 0.49 × 0.49 × 1.0 mm reconstructed voxel size); a resting-state functional MRI (rs-fMRI) using a task-free T2*-weighted blood-oxygen-level-dependent (BOLD) sequence (TR/TE 2000/29 ms, flip angle 90°, 32 slices (interleaved acquisition order), 104 × 104 matrix size, 2.0 × 2.0 × 4.0 mm reconstructed voxel size, 195 dynamic volumes); and a diffusion-tensor MRI (dMRI) using a diffusion sensitized echo-planar imaging (EPI) sequence (TR/TE 6100/57 ms, 65 slices, 100 × 100 matrix size, 64 diffusion sensitizing gradient directions (b = 1200 s/mm^2^), 2.0 mm cubic reconstructed voxel size) with three additional minimally diffusion-weighted images (b = 0 s/mm^2^). 

Contraindications for MRI assessments were the presence of a cardiac pacemaker or implantable cardioverter defibrillator, neurostimulator, nondetachable insulin pump, metallic vascular clips or stents in the head, cochlear implant, metal-containing intrauterine device, metal splinters or shrapnel, dentures with magnetic clip, an inside bracket, pregnancy, epilepsy, and claustrophobia.

### 2.3. Segmentation of Brain Tissue

T1w and FLAIR data were analyzed by use of an ISO13485:2012-certified, automated method (which included visual inspection) [31,32]. T1w data were segmented into gray matter, white matter, white matter hyperintensities (WMH), and CSF volumes (1 voxel = 1.00 mm^3^ = 0.001 mL) [31]. Intracranial volume (ICV), in which the cerebellum was included, was calculated as the sum of gray matter, white matter (including WMH volume), and CSF volumes.

### 2.4. dMRI and rs-fMRI Data Pre-Processing

dMRI as well as rs-fMRI data were first anonymized and converted from DICOM to NIfTI format using Chris Rorden’s dcm2nii tool (version 2MAY2016 64bit BSD License) for further processing.

Pre-processing of the dMRI data was mainly performed with ExploreDTI v4.8.6 (PROVIDI lab, Image Sciences Institute, Utrecht, The Netherlands) [33], and included eddy current and head motion correction [34,35], which was followed by constrained spherical deconvolution (CSD)-based deterministic whole-brain tractography [36] to obtain white matter fiber tracts. Next, the automated anatomical labeling (AAL) atlas [37], consisting of 94 (sub)cortical brain regions in the cerebrum, was (affine) coregistered to the dMRI data using FLIRT [38] in the FMRIB Software Library (FSL) 5.0.10 (FMRIB Analysis Group, University of Oxford, Oxford, UK). Lastly, for each pair of brain regions with two or more tracts running between them, the connection strength was determined as tract volume (number of voxels visited by a tract multiplied by the voxel size) relative to ICV [39,40], resulting in a symmetric 94 × 94 connectivity matrix, i.e., the participant’s structural connectome (SC), where each row and column represent a brain region and each element represents the relative tract volume between two regions.

Pre-processing of the rs-fMRI data was performed using a combination of tools in FSL 5.0.10 and Statistical Parametric Mapping (SPM) 12 (The Wellcome Trust College London, London, UK), and included magnetization stabilization followed by correction for field inhomogeneities [41], slice-timing, and head motion [42]. Next, rs-fMRI data were spatially and temporally filtered using a band-pass filter (0.01 to 0.1 Hz) to increase the signal-to-noise ratio (SNR) and remove possible respiratory and signal drift effects to focus on the spontaneous low-frequency fluctuations [12]. Lastly, the AAL atlas and individual-specific T1w including WM and CSF masks were (affine) coregistered to the rs-fMRI data using FSL’s FLIRT [38], and the average time-series for each brain region as well as for the CSF and WM were calculated from the per-voxel time-series in each region. For each pair of brain regions, the connection strength was defined as the Pearson’s correlation coefficient calculated using linear regression of the averaged time-series of each region, corrected for motion (three translational and three rotational parameters) as well as the CSF and WM signal, resulting in the participant’s functional connectome. Negative correlations, which are considered as not representing any meaningful connections, were set to zero [43].

**Figure 1:**
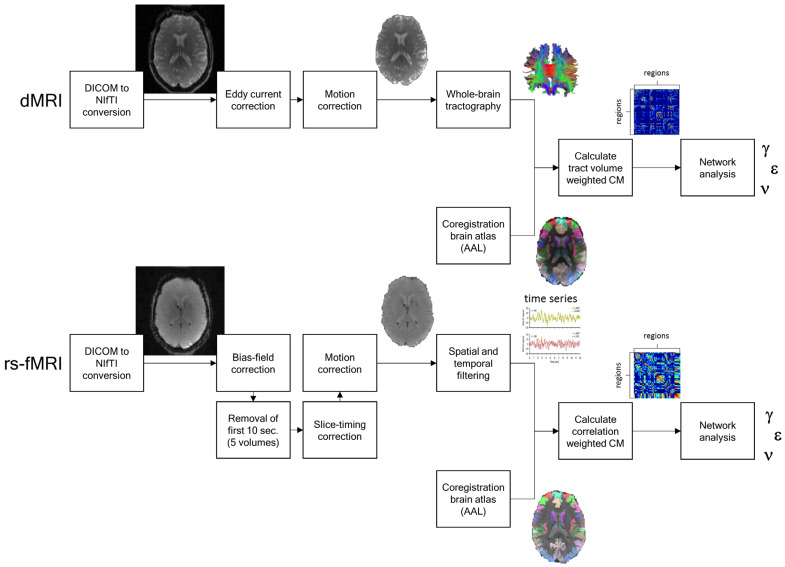
Schematic overview of the complete processing pipelines of the dMRI (**top**) and rs-fMRI (**bottom**) data to analyze structural and functional network connectivity, respectively, in terms of graph measures of average node degree (ν), normalized clustering coefficient (γ), and normalized global efficiency (ε).

In both the structural as well as the functional connectome, self–self connections, i.e., the diagonal elements, were set to zero. A complete overview of the structural and functional connectivity processing pipeline, including a description of the hardware and software, is provided in Appendix A, and a schematic overview is shown in Figure 1.

### 2.5. Brain Network Connectivity Analysis Using Graph Theory

**Figure 2:**
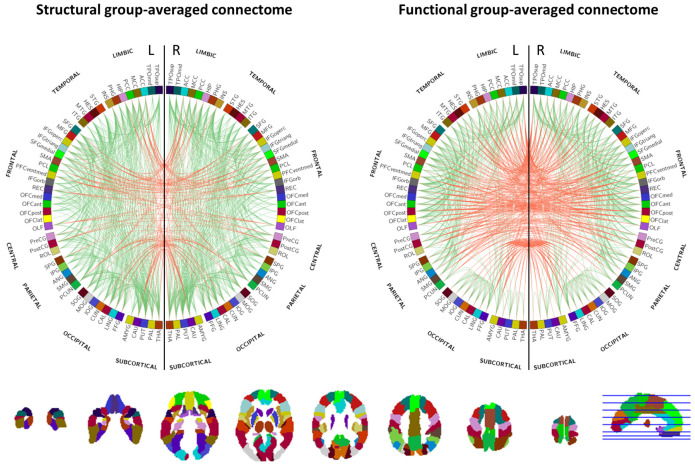
Structural (**left**) and functional (**right**) group-averaged connectomes at a sparsity of 0.80 showing the connections (number of connections = 874) between brain regions that were subsequently used in the structural and functional network connectivity analyses, respectively. (Red: interhemispheric connections, green: intrahemispheric connections). Brain region labels and colors are according to the automated anatomical labeling (AAL2) atlas which is shown underneath.

From here on, the approach to calculate the structural and functional connectivity using graph theory was similar. First, one structural and one functional group-averaged connectome were calculated from all individual structural (*n* = 5226) and functional (*n* = 5231) connectomes, respectively. For the structural group-averaged connectome, the individual connectomes were used in binarized form (relative tract volume > 0), whereas for the functional group-averaged connectome the individual connectomes were used as such. To minimize the effect of spurious connections, both group-averaged connectomes were proportionally thresholded to a default sparsity of 0.80, meaning that only the connections that were present in at least 80% of the participants were taken into account in the individual structural and functional connectivity analyses. At this sparsity of 0.80, the most contrast in connectivity measures is expected between healthy, pre-diabetic and T2DM participants [21]. A schematic representation of the structural and functional group-averaged connectomes at sparsity 0.80 is shown in Figure 2. 

Before thresholding the individual connectomes with the group-averaged connectome [44], the participant’s structural and functional overall connectivity were calculated as the mean from all weights in the SC and FC, respectively [45]. Subsequently, each participant’s connectome was masked by the group-averaged connectome, resulting in a weighted, undirected network with a sparsity close to the sparsity of the group-averaged connectome. 

From each masked individual connectome, the following theoretical network connectivity measures were calculated using graph theory: average node degree (ν), a basic global network measure that can be interpreted as the “wiring cost” of the network [46]; normalized clustering coefficient (γ), a global measure of network segregation [46,47]; and normalized global efficiency (ε_global_), a global measure of network integration [48,49]. The clustering coefficient and global efficiency were normalized to values calculated from 100 randomly generated networks of the same size, sparsity, and binary degree as the individual network [46,49]. All connectivity analyses were performed using the Brain Connectivity Toolbox [46] in MATLAB Release 2016a (The Mathworks Inc., Natick, MA, USA). 

To assess robustness of the connectivity measures over sparsity, the structural and functional group-averaged connectomes were additionally thresholded to sparsities ranging from 0.60 to 0.90 (step size 0.05) and from 0.10 to 0.90 (step size 0.10), respectively, and the connectivity measures were calculated at each of these sparsity values.

### 2.6. Quality Assessment

Uncertainty in brain connectivity measures was assessed using the following image quality metrics, each on a ‘lower is better’ scale: (1) inverse signal-to-noise ratio (iSNR) of the unprocessed images, (2) amount of head motion, and (3) spatial mismatch between the pre-processed dMRI or rs-fMRI data and the AAL brain atlas:

- Inverse signal-to-noise ratio

The iSNR [-] was calculated according to Equation (1) [50]:

(1) $$\mathrm{iSNR}=-\frac{\mathrm{mean}\left(I_{1},I_{2}\right)}{\mathrm{std}\left(I_{1}-I_{2}\right)/\mathrm{sqrt}\left(2\right)}$$

where *I*_1_ and *I*_2_ were two (brain masked) volumes that were acquired immediately after each other at b = 0 s/mm^2^ at the end of the dMRI scan. For the rs-fMRI scan, *I*_1_ and *I*_2_ were the first two volumes that were acquired after removal of the first 10 s to account for magnetic stabilization, i.e., the 5th and 6th volume.

- Head motion

The amount of head motion was expressed as mean volume-to-volume translation, which was calculated from the translational parameters from the rigid body correction for head motion according to Equation (2) [24]. In short, the translational head motion [mm] of a volume was computed as the root-mean-square of displacements in the sagittal, coronal, and transverse planes:

(2) $$\mathrm{Translation}=\sum_{i=2}^{N}\frac{\sqrt{{\left(X_{i}-X_{i-1}\right)}^{2}+{\left(Y_{i}-Y_{i-1}\right)}^{2}+{\left(Z_{i}-Z_{i-1}\right)}^{2}}}{N-1}$$

where *N* is the number of volumes in the dMRI or rs-fMRI data; and *X*, *Y*, and *Z* are the displacements of the *i*^th^ volume along the left–right, anterior–posterior, and longitudinal axes, respectively.

- Mismatch between brain atlas and pre-processed data

The spatial mismatch between the pre-processed dMRI or rs-fMRI data and the AAL brain atlas was quantified using 1-Dice’s similarity coefficient [51] according to Equation (3):

(3) $${\mathrm{Mismatch}}_{\mathrm{atlas}}=1-\frac{2\left|A\cap B\right|}{\left|A\right|+\left|B\right|}$$

where *A* is the number of voxels in the brain mask of the dMRI or rs-fMRI data and *B* is the number of voxels in the brain mask of the AAL template. Mismatch varies between 0 and 1 representing no and complete mismatch, respectively.

### 2.7. Demographic and Clinical Variables

Demographic and clinical data were collected as previously described [9]. Variables of interest included age, sex, body mass index (BMI), and history of cardiovascular disease (‘No’, or ‘Yes’). Educational level was assessed by interview and classified into eight levels commonly used in the Netherlands: (1) no education, (2) primary education, (3) lower vocational education, (4) intermediate general secondary education, (5) intermediate vocational education, (6) higher general secondary education, (7) higher vocational education, and (8) university degree. For this study, educational level was divided into three groups: Low (levels 1–3), Middle (levels 4–6), and High (levels 7 and 8). Based on their glucose metabolism status as determined according to the World Health Organization’s criteria by a 75 g two-hour glucose tolerance test (OGTT) after an overnight fast [52], participants were categorized into either ‘No diabetes’ (normal glucose metabolism), ‘Prediabetes’, ‘Type 2 diabetes’, or ‘Other type of diabetes’ [9]. 

### 2.8. Statistics

Structural and functional brain connectivity measures were reported using the appropriate descriptive statistics, e.g., means and standard deviation in the case of normally distributed data, median and 25–75th percentiles for non-normally distributed data, or percentages for categorical data. Correlations between dMRI and rs-fMRI quality metrics were assessed using Pearson’s correlation. Multiple linear regression was used to assess the relationship between quality metrics and structural and functional connectivity measures. 

To study the effect of image quality on the association between brain connectivity and demographic and clinical variables, two linear regression models were used. In Model 1, the connectivity measure was the dependent variable and age, sex, BMI, diabetes status, educational level, history of CVD, and WMH volume were the independent variables. In Model 2, we additionally adjusted for the image quality metrics. Skewed variables (WMH volume) were log10-transformed. Significant regression coefficients that changed more than 10% were considered as relevant changes.

To ascertain which of the demographic and clinical variables of age, sex, BMI, diabetes status, educational level, history of CVD, and WMH volume were associated with the quality metrics, linear regression was used. Skewed variables (WMH volume) were log10-transformed.

All statistical analyses used a level of significance of 0.05, and were performed in IBM SPSS Statistics for Windows, version 25 (IBM Corp., Armonk, NY, USA). 

## 3. Results

Demographic and clinical characteristics, brain connectivity estimates at a sparsity of 0.80, and dMRI and rs-fMRI image quality metrics in the participants that were included in this study (*n* = 5110) are listed in Table 1.

**Figure A2:**
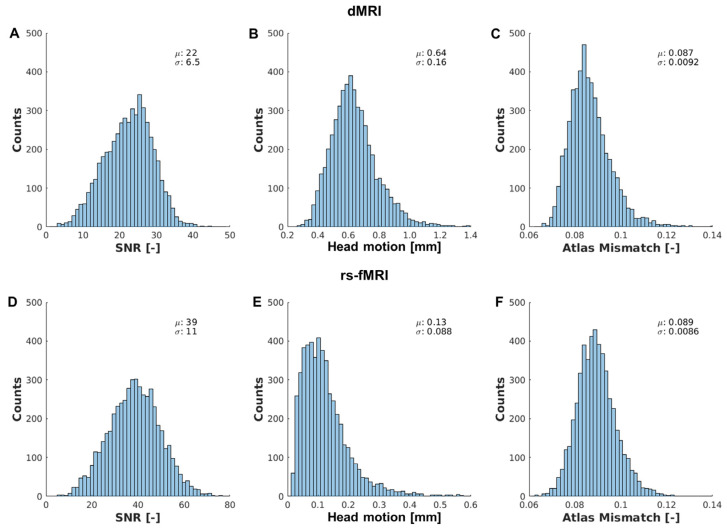
Histograms of dMRI (**top**) and rs-fMRI (**bottom**) image quality metrics of signal-to-noise ratio (SNR) (**A**,**D**), amount of head motion (**B**,**E**), and atlas mismatch (**C**,**F**).

Histograms of dMRI and rs-fMRI image quality metrics SNR, head motion, and atlas mismatch are reported in Figure A2. For both dMRI as well as rs-fMRI, the strongest correlation was observed between the quality metrics iSNR and head motion (r = 0.298 and r = 0.522, respectively, both *p* < 0.001). A cross-table reporting the Pearson’s correlation coefficients between all dMRI and rs-fMRI quality metrics is given in Table A2.

**Figure A3:**
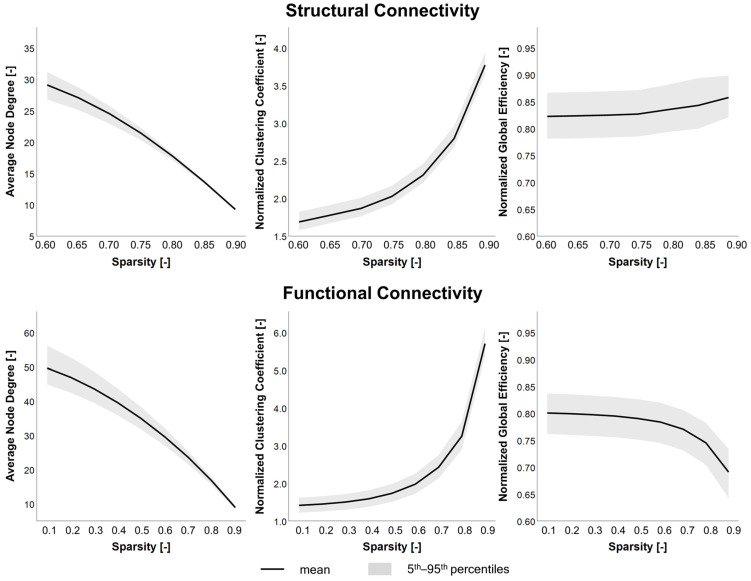
Mean and 5–95th percentiles of structural (**top**) and functional (**bottom**) connectivity measures of ν (**left**), γ (**middle**), and ε_global_ (**right**) over the range of sparsities.

Mean and 5–95th percentiles of structural and functional connectivity measures ν, γ, and ε_global_ are plotted over the range of sparsities in Figure A3. Mean (standard deviation (SD)) of overall structural and functional connectivity (note that these are sparsity-independent) were 5.3 * 10^−3^ (0.6 * 10^−3^) and 0.32 (0.03), respectively.

### 3.1. Associations of Connectivity Measures with dMRI and rs-fMRI Quality Metrics

**Figure 3:**
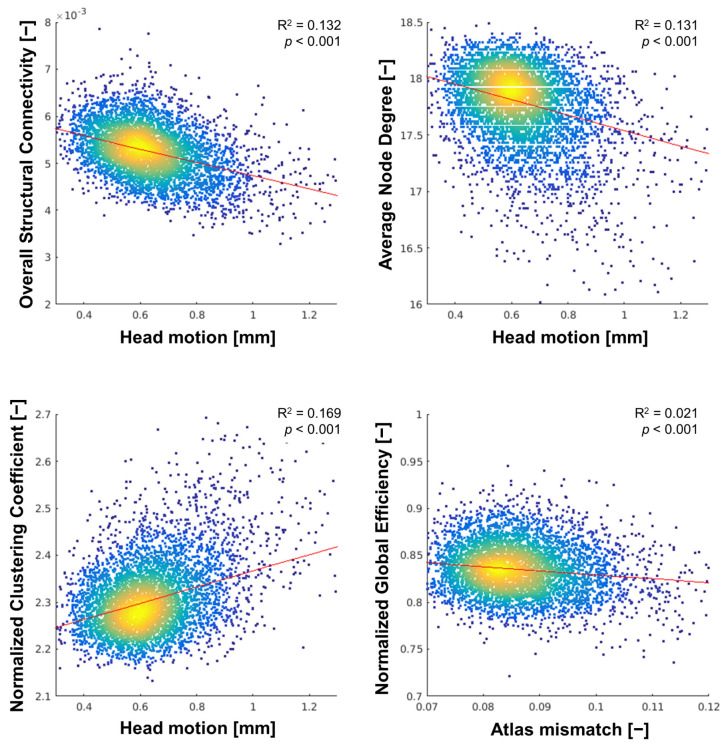
Density scatterplots of structural connectivity measures at sparsity 0.80 vs. the quality metrics with the strongest association.

The diffusion MR image quality metrics iSNR, head motion, and atlas mismatch were all related to structural connectivity measures of overall SC, ν, and γ, with the strongest associations for head motion with standardized regression coefficients (β) ranging from –0.36 to 0.40 (all *p* < 0.001), while atlas mismatch was most strongly related to ε_global_ (β = –0.15, *p* < 0.001), as shown in Figure 3. A full overview of the associations between the diffusion and functional MR image quality metrics and the structural and functional connectivity measures, respectively, as well as the R^2^ for each model, is reported in Appendix C, Table A3A,B.

**Figure 4:**
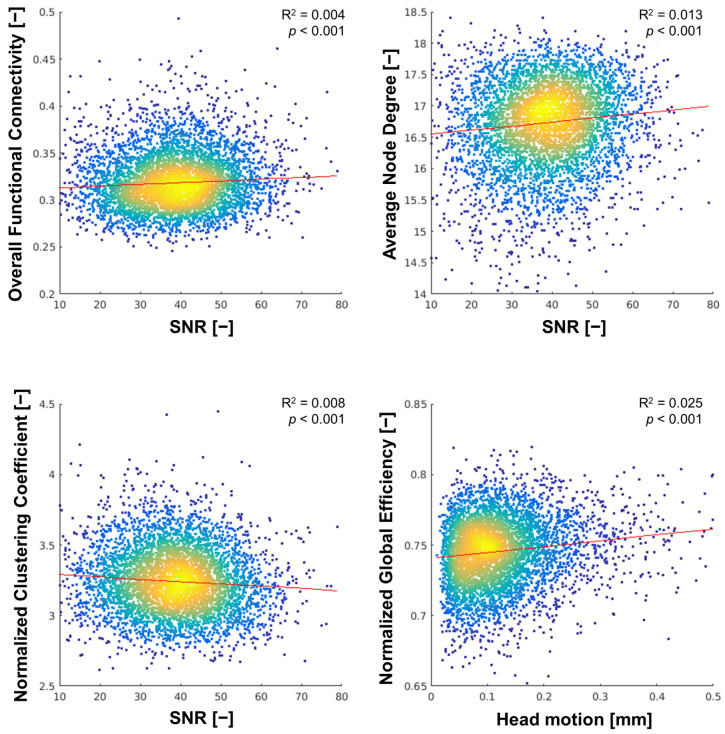
Density scatterplots of functional connectivity measures at sparsity 0.80 vs. the quality metrics with the strongest association. Note: for intuitiveness, SNR (expressed as -iSNR) is plotted instead of iSNR.

From the functional MR image quality metrics, iSNR was most strongly related to each of the functional connectivity measures of overall FC, ν, and γ, with standardized regression coefficients (β) ranging from –0.22 to 0.15 (all *p* < 0.001), except for ε_global_, for which head motion had the strongest association (β = 0.16, *p* < 0.001), as shown in Figure 4. 

The variability in the SC measures was consistently better explained by the quality metrics than variability in the FC measures, with R^2^ values ranging from 0.030 to 0.173 (3.0% to 17.3%) for the SC measures (see Appendix C Table A3A) and 0.006 to 0.032 (0.6% to 3.2%) for the FC measures (see Appendix C Table A3B). 

### 3.2. Effect of dMRI and rs-fMRI Quality on Connectivity Associations 

Standardized regression coefficients (β) of the regression model between the structural or functional connectivity measures and the demographic/clinical variables, and the same model with additional adjustment for the image quality metrics are shown in Table 2 and Table 3. 

Without adjustment for image quality, in particular higher age and male sex were associated with lower overall structural connectivity (β = −0.180, *p* < 0.001; and β = 0.284, *p* < 0.001, respectively), lower average node degree (β = −0.222, *p* < 0.001; and β = 0.058, *p* < 0.001, respectively), and higher clustering coefficient (β = 0.179, *p* < 0.001; and β = −0.207, *p* < 0.001, respectively). With adjustment for diffusion MR image quality, the aforementioned associations with age and sex decreased by more than 26%. Without adjustment for image quality, higher age, but not sex, was associated with higher global efficiency (β = 0.171, *p* < 0.001; and β = 0.018, *p* = 0.204, respectively), while male sex was found to be associated with lower global efficiency (β = −0.076, *p* < 0.001) after adjustment for image quality. Of note, significant associations were also observed for different combinations of demographic/clinical variables and the structural connectivity measures, with standardized regression coefficients (β) generally <0.1 with relevant changes (>10%) after adjustment for image quality (See Table 2).

Without adjustment for image quality, higher age and male sex were associated with lower functional average node degree (β = −0.129, *p* < 0.001) and higher functional global efficiency (β = −0.113, *p* < 0.001), respectively, and these associations did not change after adjustment for functional MR image quality. No other associations with |β| > 0.1 were observed between the demographic/clinical variables and the functional connectivity measures (see Table 3).

### 3.3. Associations between Quality and Demographic Variables

Standardized regression coefficients (β) between the demographic/clinical variables and each of the diffusion and functional MR image quality metrics, including the R^2^ value for the complete model, are listed in Table 4 and Table 5. 

From the three diffusion MR image quality metrics (Table 4), variance in head motion could be best explained by the demographic/clinical variables (R^2^ = 0.297) with age (β = 0.368, *p* < 0.001) and sex (β = −0.279, *p* < 0.001), indicating more head motion at higher age and in men compared to women, as the strongest covariates. Mismatch between diffusion MRI and brain atlas had the strongest association with sex (β = −0.303, *p* < 0.001), indicating less mismatch in women compared to men. Conversely, the amount of variance in the iSNR of the diffusion MRI that could be explained by the demographic/clinical variables was negligible (R^2^ = 0.033). 

**Figure 5:**
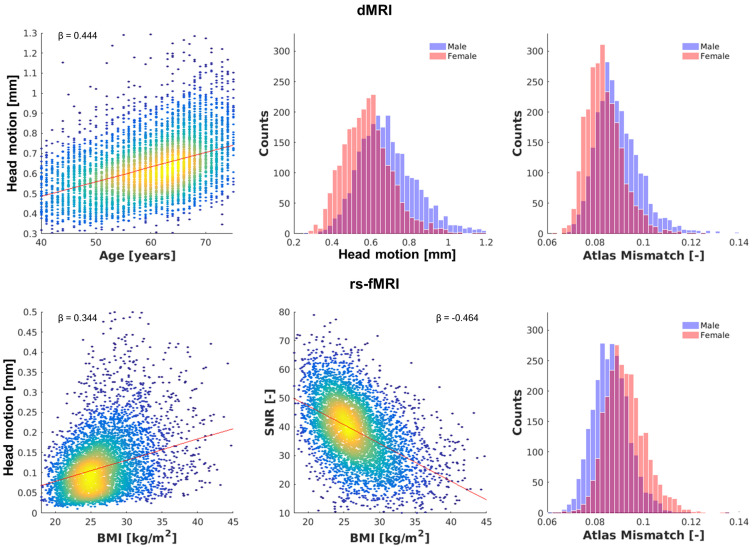
Examples of strongest determinants of quality metrics in the structural (**top**) and functional (**bottom**) connectivity pipeline. For structural quality, motion was best determined by age and sex, and atlas mismatch by sex. For functional quality, motion and iSNR were best determined by BMI, and atlas mismatch by sex. Note: for intuitiveness, SNR (expressed as -iSNR) is plotted instead of iSNR.

From the three functional MR image quality metrics (Table 5), variance in iSNR could be best explained by the demographic/clinical variables (R^2^ = 0.270) with BMI (β = 0.408, *p* < 0.001) as the strongest covariate. Variance in head motion and atlas mismatch of the functional MRI were best explained by BMI (β = 0.321, *p* < 0.001) and sex (β = 0.317, *p* < 0.001), respectively. Scatterplots and histograms visualizing the strongest associations between quality metric and demographic variables are shown in Figure 5.

## 4. Discussion

### 4.1. Main Findings 

We extensively studied the association of dMRI and rs-fMRI quality with structural and functional connectivity measures, respectively, in 5110 participants of The Maastricht Study. To summarize, we found a significant association between the dMRI and rs-fMRI quality metrics, i.e., head motion and signal-to-noise ratio in particular, respectively, and measures of structural and functional connectivity. Moreover, the image quality metrics affected the association between brain connectivity measures and demographic variables. Furthermore, our results showed that the image quality metrics were equally or even stronger determinants of brain structural and functional connectivity than demographic and/or clinical variables. 

### 4.2. Head Motion

Head motion during the dMRI scan was most strongly associated with three of the four structural connectivity measures studied here, indicating it is an important potential confounder. To put the effect of head motion into perspective, every 0.1 millimeter of head motion during dMRI can be misinterpreted as a decrease in overall structural brain connectivity similar to 18.3 years of aging (see Appendix D for derivation). 

For dMRI as well as rs-fMRI, the amount of head motion increased with age and was larger in men compared to women. These results are in line with the current literature, as similar findings have been reported earlier [22,24,53,54]. In addition, the amount of head motion during the rs-fMRI scan increased with BMI, confirming the findings reported earlier [55,56], and might be caused by the larger respiration-related body displacement in persons with high BMI. 

We also found that the amount of head motion was larger in the dMRI compared to the rs-fMRI scan (mean head motion 0.64 mm and 0.13 mm, respectively), and that they were only weakly correlated (Pearson’s r = 0.21). An explanation for this finding might lie in the nature of the pulse sequences, for instance the longer echo and repetition times and the much stronger gradients of the dMRI scan, leading to notable table vibrations and head coil vibrations, which may amplify any distorting effects due to head motion compared to the rs-fMRI scan used. Furthermore, the dMRI sequence was applied after the rs-fMRI sequence, at the end of the scan protocol. Hence, assuming that participants are more likely to move with longer scan times, this might explain the higher amount of head motion during the dMRI compared to the rs-fMRI. 

To assess whether the observed effects are robust to large motion, we performed a post hoc analysis in which the cases with motion > 1.0 mm were excluded (n = 121 excluded). The results (see Appendix E, Table A4, Table A5, Table A6 and Table A7) showed that excluding the cases with the strongest motion only marginally affected the results, and that the interpretation of the results and conclusions that were drawn from these results do not change.

### 4.3. Brain Atlas Mismatch

Although the distribution of atlas mismatches in the studied population is highly comparable for dMRI and rs-fMRI (Figure A2C,F), atlas mismatch was associated with three out of four structural connectivity measures, but not with any of the functional connectivity measures. However, the dMRI-based structural connectivity measures rely on the geometric start and end (voxel) points as well as trajectories of streamlines connecting these points, which are likely more susceptible to geometric distortions than the rs-fMRI-based functional connectivity measures, which are based on spatially region-averaged signal time-series. 

Since atlas mismatch and head motion are both associated with three out of four structural connectivity measures, they might have a common source, i.e., typical susceptibility artifacts due to the EPI sequences used during dMRI that are known to be highly prone to resonance offsets, e.g., magnetic susceptibility gradients, or B0 inhomogeneities [57], for which ExploreDTI did not correct. The linear registration that we used to co-register the atlas to dMRI space is only able to account for deformations caused by these susceptibility artefacts to a limited extent. Whether the use of a non-linear registration procedure, an individual-based atlas, or implementation of a more rigorous susceptibility correction method will lead to less mismatch was beyond the scope of the current study.

### 4.4. Signal-to-Noise Ratio (SNR)

SNR was weakly associated with one structural connectivity measure, i.e., overall SC, but with three out of four functional connectivity measures, indicating that, in addition to head motion, SNR is a quality metric of interest in functional brain connectivity analyses. Interestingly, SNR, as well as head motion in rs-fMRI, and to a lesser extent also in dMRI, decreased with BMI, which might have a physiological explanation as respiratory function is altered in obesity [58], especially when scanned in the supine position, which may increase physiological-related noise [59]. Since the SNR and head motion in rs-fMRI are moderately correlated (Pearson’s r = −0.52, *p* < 0.001), which is comparable to the results reported by Van Dijk et al. (r = −0.57, *p* < 0.001) [24], but the amount of head motion is relatively small (mean = 0.13 mm), we propose that the small amount of head motion is propagated into the SNR.

### 4.5. Effect of Image Quality on Associations between Brain Connectivity and Demographic Variables

Since the image quality metrics were significantly related to measures of structural and functional connectivity, it is apparent that they affect the associations between structural or functional connectivity and demographic/clinical variables. Indeed, without adjustment for image quality, the strength of the associations between structural connectivity and demographic variables, particularly age and sex and to a lesser extent BMI and WMH volume, differed by more than 25% compared to the model that adjusted for image quality. Interestingly, whereas the age- and sex-related associations with brain connectivity were weakened due to confounding effects of image quality, BMI–brain connectivity associations were actually strengthened when image quality was taken into account. A plausible explanation for this observation is currently still lacking. Although the ground truth structural connectivity in our study sample is unknown, the fact that age-, sex-, and BMI-related associations are affected by image quality underlines the importance of adjusting for it.

For functional connectivity associations with demographic/clinical variables, however, the changes due to adjustment for image quality were much smaller. An explanation for this discrepancy is that the gradients in dMRI are stronger than in rs-fMRI, hence any artifact is more pronounced in the dMRI and thus the effect of low image quality is stronger. Moreover, during the pre-processing of the rs-fMRI data, head motion is already taken into account by adding the motion parameters as nuisance regressors to the regression model when calculating temporal correlation between two brain regions. 

### 4.6. Validity of Structural and Functional Connectivity Results

Analyses of the connectivity associations with demographic/clinical variables demonstrated that overall structural and functional connectivity, and hence node degree, decrease with age, whereas normalized clustering coefficient and global efficiency increase with age. This finding suggests that despite decreasing connectivity, whole-brain network segregation and integration increase during aging. Decreased structural and functional connectivity during aging is consistent with the current consensus as summarized in a recent review [60]. Less consensus, however, exists in the literature on the association of global efficiency with age. For example, structural and functional global efficiency were lower in older people compared to young people [61,62], or showed no difference between old and young people [63,64,65], whereas we found a slight positive association. The positive association between the structural clustering coefficient and age that we found confirms the findings reported by Zhao et al. [62]. Yet, it has to be noted that the aforementioned findings have been reported in studies with relatively small sample sizes (n ≤ 126) compared to our study. 

The validity of our structural and functional connectivity results is further supported by their dependency on sparsity. Both the structural and functional average node degree decreased with sparsity. This was as expected, since with increasing sparsity fewer connections are evaluated, and thus the number of possible connections to each node decreases as well. Structural and functional normalized clustering coefficient increased with sparsity. This effect, too, can be explained by the methodology used, because the proportion of connections between the nodes within its neighborhood divided by the number of connections that theoretically could exist between them will decrease with increasing sparsity. Conversely, the normalized clustering coefficient increases at increasing sparsity, because the clustering coefficient is normalized to a random network, for which the proportional decrease is larger. 

**Figure A4:**
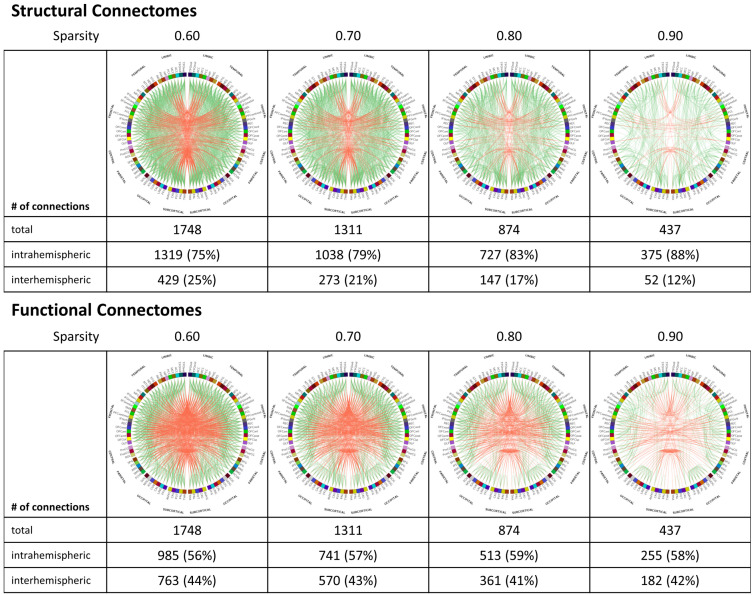
Structural (**top**) and functional (**bottom**) group-averaged connectomes at sparsity 0.60 to 0.90. Whereas the ratio of intra- and interhemispheric connections remains fairly stable for the functional connectomes, this ratio increases rapidly for the structural connectomes.

In contrast, the normalized global efficiency over sparsity showed an opposite trend in the structural compared to the functional connectomes. This difference can be explained by varying number of intra- and interhemispheric connections taken into account in the structural and functional group-averaged connectomes over the sparsity range. While the percentage of interhemispheric connections in the functional group-averaged connectomes remains fairly constant (at 41–44%), this percentage decreases in the structural group-averaged connectomes from 25% at sparsity of 0.60 to 12% at sparsity of 0.90 (see Figure A4). As the connection weights in the structural connectomes represent tract volumes, and since the structural connections taken into account are mostly short (intra-hemispheric) tracts with a small volume, the structural global efficiency is calculated using fairly low connection strengths that increases with sparsity, whereas the functional global efficiency is based on connections of high strength that increase with sparsity.

The result that fewer interhemispheric connections were taken into account in the structural compared to the functional connectomes can be explained by the effect of length and shape of the tracts in whole-brain tractography [66]. Since interhemispheric tracts are generally longer than intrahemispheric tracts, they are more difficult to track and are thus less likely to end up in the group-averaged connectome in favor of intrahemispheric connections.

### 4.7. Strengths and Limitations

Strengths of this study include the large number of participants and the acquisition of dMRI as well as rs-fMRI data in these participants. However, there are also several limitations that are noteworthy to address. First of all, due to missing field maps, we could not implement any advanced correction for B0-field inhomogeneities or geometric distortions, e.g., FSL’s “topup” [67], in the dMRI and rs-fMRI processing pipelines. Hence, we were restricted to less advanced methods, such as (affine) registration to standard space. Consequently, the results relating to regions in the anterior frontal cortex and temporal lobe might therefore be less reliable as geometric distortions often occur in this location [68]. However, to our knowledge, there is no reason to assume that these artifacts differ between subgroups, e.g., participants with and without T2DM, and therefore no bias has been introduced. 

Second, we used an atlas template that is not participant-specific and as such may contribute to mismatch between the brain regions defined in the atlas and the actual functional boundaries of the brain regions in the participant’s dMRI or rs-fMRI. An individual-based brain parcellation, such as implemented in the FreeSurfer software [69], might result in better overlap with the participant’s dMRI or rs-fMRI. However, this requires substantially longer processing times, e.g., up to 20 h per participant, as well as visual checks and manual intervention, whereas linear registration of the AAL2 atlas is robust and is typically completed within a minute.

Third, we did not compare different structural and functional connectivity processing pipelines in this study to assess the most suitable pipelines. Since systematic variability across connectivity pipelines can yield misleading results, as shown recently by Luppi et al. [70], further optimization of the processing pipelines might be warranted. Nevertheless, we believe we obtained valid structural and functional connectivity results, as discussed in the previous section.

Third, due to the intrinsic differences in dMRI and rs-fMRI sequences, direct comparison between their image quality does not seem valid. However, during setup of the scanning protocol, both sequences were considered equally important, i.e., both were optimized in terms of acquisition time, signal-to-noise ratio, and spatial resolution without sacrificing one sequence over the other. 

Lastly, by using full correlation to calculate functional connectivity, our processing pipeline might be less sensitive to certain confounding effects, e.g., global mean confounding. Alternative approaches, such as partial correlation, regularized inverse covariance, or Bayes net methods, can improve the sensitivity of the functional connectivity processing pipeline [43].

## 5. Conclusions

To conclude, we here describe the complete pipeline analyses for the assessment of the structural and functional brain connectivity in The Maastricht Study, including extensive quality assessment focused on the confounding effects of compromised image quality in population neuroimaging studies. Structural connectivity estimates were most strongly associated with head motion, while functional connectivity estimates were mainly influenced by signal-to-noise ratio, possibly resulting from motion as well, especially in patients with high BMI. Moreover, image quality metrics substantially affected the associations between brain connectivity and demographic and clinical variables such as age, sex, and BMI. These results largely confirm and complement previously reported findings and we therefore recommend that statistical analyses of structural and functional brain connectivity and its associations with demographic or clinical variables should report and consider potential confounding effects of image quality.

## Figures and Tables

**Table 1:** Characteristics of participants with successfully processed dMRI and rs-fMRI data (*n* = 5110).

Characteristic	
*Demographic*	
Age [mean (SD), years]	59.4 (8.7)
Sex [%] Male Female	50.6 49.4
Educational level [%] ^†^ Low Medium High	32.0 28.4 39.7
*Clinical*	
BMI [mean (SD), kg/m^2^]	26.6 (4.2)
Diabetes status [%] No diabetes Prediabetes Type 2 diabetes Other type of diabetes	64.1 14.7 20.6 0.6
History of CVD [%] ^‡^ No Yes	87.4 12.6
Relative WMH volume [median (25^—^75th percentile), % of ICV]	0.016 (0.005–0.050)
*Brain connectivity*	
Structural connectivity [mean (SD), -] Overall Average node degree, ν Clustering coefficient, γ Global efficiency, ε_global_	5.3 * 10^−3^ (0.6 * 10^−3^) 17.8 (0.4) 2.31 (0.08) 0.84 (0.03)
Functional connectivity [mean (SD), -] Overall Average node degree, ν Clustering coefficient, γ Global efficiency, ε_global_	0.32 (0.03) 16.7 (0.7) 3.25 (0.23) 0.75 (0.02)
*Image quality*	
dMRI Signal-to-noise ratio [mean (SD), -] Head motion [mean (SD), mm] Atlas mismatch [mean (SD), -]	22 (6.5) 0.64 (0.16) 0.087 (0.0092)
rs-fMRI Signal-to-noise ratio [mean (SD), -] Head motion [mean (SD), mm] Atlas mismatch [mean (SD), -]	39 (11) 0.13 (0.088) 0.089 (0.0086)

Abbreviations: SD: standard deviation; BMI: body mass index; CVD: cardiovascular disease; WMH: white matter hyperintensities; ICV: intracranial volume; dMRI: diffusion-weighted magnetic resonance imaging; rs-fMRI: resting-state functional magnetic resonance imaging. Missing data: ^†^ Educational level (N = 62); ^‡^ History of CVD (N = 57).

**Table 2:** Standardized regression coefficients (β) of the regression model between the structural connectivity measures at sparsity 0.80 and the demographic/clinical variables (Model 1), and the same model with additional adjustment for the diffusion MR image quality metrics (Model 2). For significant regression coefficients, the percentage change is also reported.

Independent Variables	Overall SC			ν			γ			ε_global_		
	Model 1	Model 2	∆ [%]	Model 1	Model 2	∆ [%]	Model 1	Model 2	∆ [%]	Model 1	Model 2	∆ [%]
Age	−0.179 ***	−0.074 ***	−59	−0.222 ***	−0.110 ***	−50	0.179 ***	0.059 ***	−67	0.171 ***	0.180 ***	+5
Sex	0.284 ***	0.213 ***	−25	0.058 ***	−0.081 ***	−240	−0.207 ***	−0.096 ***	−54	−0.018	–0.076 ***	+322
Educational level	−0.044 **	−0.045 ***	+2	0.025	0.024	-	–0.007	−0.007	-	−0.010	–0.011	-
BMI	0.096 ***	0.109 ***	+14	0.009	0.035 *	+289	−0.037 *	−0.054 ***	+46	−0.013	0.002	-
Diabetes status	−0.025	0.003	-	−0.103 ***	−0.082 ***	−20	0.074 ***	0.046 **	–38	0.015	0.010	-
History of CVD	−0.015	–0.010	-	−0.034 *	−0.027 *	−21	0.021	0.014	-	−0.010	−0.008	-
WMH volume ^†^	0.041 **	0.048 ***	+17	−0.053 ***	−0.036 **	−32	0.093 ***	0.077 ***	−17	−0.013	−0.008	-
iSNR	-	−0.120 ***	-	-	0.008	-	-	0.006	-	-	0.047 **	-
Head motion	-	−0.249 ***	-	-	−0.276 ***	-	-	0.310 ***	-	-	−0.008	-
Atlas mismatch	-	0.021	-	-	−0.206 ***	-	-	0.081 ***	-	-	−0.194 ***	-
**R^2^**	0.131	0.204	+56	0.097	0.197	+103	0.121	0.199	+64	0.031	0.065	+110

Models: (1) Connectivity measure = β_0_ + β_1_ * Age + β_2_ * Sex + β_3_ * Educational level + β_4_ * BMI + β_5_ * Diabetes status + β_6_ * History of CVD + β_7_*WMH volume. (2) Connectivity measure = Model 1 + β_8_ * iSNR + β_9_ * Head motion + β_10_ * Atlas mismatch. Abbreviations: BMI: body mass index; CVD: cardiovascular disease; WMH: white matter hyperintensity volume; iSNR: inverse signal-to-noise ratio. ^†^ Log^10^-transformed. Significant at * *p* < 0.05; ** *p* < 0.01; *** *p* < 0.001. Significant regression coefficients that changed more than 10% are expressed in bold.

**Table 3:** Standardized regression coefficients (β) of the regression model between the functional connectivity measures at sparsity 0.80 and the demographic/clinical variables (Model 1), and the same model with additional adjustment for the functional MR image quality metrics (Model 2). For significant regression coefficients, also the percentage change is calculated.

Independent Variables	Overall FC			ν			γ			ε_global_		
	Model 1	Model 2	∆ [%]	Model 1	Model 2	∆ [%]	Model 1	Model 2	∆ [%]	Model 1	Model 2	∆ [%]
Age	−0.003	0.015	-	−0.129 ***	−0.119 ***	−8	0.050 **	0.042 **	−16	0.063 ***	0.050 **	−21
Sex	−0.054 **	−0.062 ***	+15	0.006	0.003	-	−0.006	−0.012	-	−0.113 ***	−0.095 ***	−16
Educational level	0.017	0.014	-	0.039 **	0.036 *	−8	−0.045 **	−0.042 **	−7	0.013	0.015	-
BMI	−0.029	−0.008	-	−0.030 *	**−0.005**	−83	0.001	−0.017	-	0.094 ***	0.049 **	−48
Diabetes status	−0.037 *	**−0.030**	-	−0.065 ***	−0.060 ***	−8	0.060 ***	0.054 ***	−10	0.013	0.008	-
History of CVD	−0.016	−0.017	-	−0.040 **	−0.041 **	+3	0.009	0.010	-	0.010	0.011	-
WMH volume ^†^	−0.030 *	**−0.027**	-	−0.058 ***	−0.057 ***	−2	0.021	0.018	-	0.000	0.002	-
iSNR	-	−0.082 ***	-	-	−0.052 **	-	-	0.075 ***	-	-	0.004	-
Head motion	-	0.048 **	-	-	−0.010	-	-	−0.035 *	-	-	0.127 ***	-
Atlas mismatch	-	0.027	-	-	0.004	-	-	0.016	-	-	−0.029	-
**R^2^**	0.007	0.012	+71	0.046	0.049	+7	0.013	0.017	+31	0.033	0.048	+45

Models: (1) Connectivity measure = β_0_ + β_1_ * Age + β_2_ * Sex + β_3_ * Educational level + β_4_ * BMI + β_5_ * Diabetes status + β_6_ * History of CVD + β_7_*WMH volume. (2) Connectivity measure = Model 1 + β_8_ * iSNR + β_9_ * Head motion + β_10_ * Atlas mismatch. Abbreviations: BMI: body mass index; CVD: cardiovascular disease; WMH: white matter hyperintensity volume; iSNR: inverse signal-to-noise ratio. ^†^ Log^10^-transformed. Significant at * *p* < 0.05; ** *p* < 0.01; *** *p* < 0.001. Significant regression coefficients that changed more than 10% are expressed in bold.

**Table 4:** Standardized regression coefficients (β) obtained from a linear regression model with forward selection between demographic/clinical variables and each of the quality metrics for the diffusion MRI.

Independent Variables	iSNR		Head Motion		Atlas Mismatch	
	β	*p*-Value	β	*p*-Value	β	*p*-Value
Age	0.117	<0.001	0.368	<0.001	0.056	<0.001
Sex	−0.071	<0.001	−0.279	<0.001	−0.303	<0.001
Educational level	−0.017	0.242	0.001	0.916	−0.009	0.519
BMI	0.067	<0.001	0.028	0.031	0.093	<0.001
Diabetes status	0.032	0.042	0.095	<0.001	−0.023	0.129
History of CVD	0.004	0.774	0.020	0.093	0.009	0.514
WMH volume ^†^	−0.038	0.009	0.048	<0.001	0.017	0.224
**R^2^**	0.033		0.297		0.113	

Abbreviations: BMI: body mass index; CVD: cardiovascular disease; WMH: white matter hyperintensity volume; iSNR: inverse signal-to-noise ratio. ^†^ Log^10^-transformed.

**Table 5:** Standardized regression coefficients (β) obtained from a linear regression model with forward selection between demographic/clinical variables and each of the quality metrics for the functional MRI.

Independent Variables	iSNR		Head Motion		Atlas Mismatch	
	β	*p*-Value	β	*p*-Value	β	*p*-Value
Age	0.170	<0.001	0.079	<0.001	−0.088	<0.001
Sex	−0.027	0.034	−0.071	<0.001	0.317	<0.001
Educational level	−0.044	<0.001	−0.010	0.457	−0.003	0.847
BMI	0.408	<0.001	0.321	<0.001	−0.113	<0.001
Diabetes status	0.096	<0.001	0.024	0.100	−0.020	0.172
History of CVD	−0.012	0.315	−0.004	0.784	0.004	0.772
WMH volume ^†^	0.030	0.015	−0.016	0.249	0.013	0.337
**R^2^**	0.270		0.132		0.142	

Abbreviations: BMI: body mass index; CVD: cardiovascular disease; WMH: white matter hyperintensity volume; iSNR: inverse signal-to-noise ratio. ^†^ Log^10^-transformed.

## Data Availability

The Maastricht Study data dictionary is available open access via the following link: https://demaastrichtstudie.app/data-dictionary.

## Appendix A. Complete Description of Structural and Functional Connectivity Processing Pipeline

### Appendix A.1. Diffusion Data Pre-Processing

dMRI data were anonymized and converted from DICOM to NIfTI format first using Chris Rorden’s dcm2nii tool (version 2MAY2016 64bit BSD License). After importing the NIfTI files into ExploreDTI v4.8.6 (PROVIDI lab, Image Sciences Institute, Utrecht, The Netherlands) [33], eddy current and head motion correction was applied, while making sure the b-matrix was rotated accordingly [34,35]. Next, white matter tracts were calculated using a constrained spherical deconvolution (CSD)-based deterministic tractography algorithm [36] at the following settings: 2 mm seed point resolution with seed points placed randomly throughout the whole brain; step size 1 mm; and maximum harmonic degree of 8 [71]. Stopping criteria were as follows: fiber orientation distribution < 0.1; angle deviation > 30°; fibers leaving the brain mask; or fiber length < 50 mm or > 500 mm.

The automated anatomical labeling (AAL) atlas [37], consisting of 94 (sub)cortical brain regions in the cerebrum, was (affine) coregistered to the dMRI data using FLIRT [38] in FMRIB Software Library (FSL) 5.0.10 (FMRIB Analysis Group, University of Oxford, Oxford, UK) and imported into ExploreDTI.

Subsequently, for each pair of brain regions, the connection strength was defined as the tract volume (number of voxels visited by a tract multiplied by the voxel size) divided by the ICV if two or more tracts were found between the two brain regions; otherwise, the connection was considered as absent and the connection strength set to zero [39]. Also, the diagonal elements (i.e., self–self connections) were set to zero. Finally, this resulted in a symmetric 94 × 94 connectivity matrix, i.e., the participant’s structural connectome (SC), were each row and column represents a brain region and each element represents the relative tract volume between two regions.

### Appendix A.2. Functional Data Pre-Processing

The rs-fMRI data were anonymized and converted from DICOM to NIfTI format using Chris Rorden’s dcm2nii tool (version 2MAY2016 64bit BSD License). To account for magnetization stabilization, the first ten seconds of data (equivalent to the first five volumes) were removed, and the remaining rs-fMRI volumes were corrected for field inhomogeneities using FSL 5.0.10 (FMRIB Analysis Group, University of Oxford, Oxford, U.K.) [41]. The rs-fMRI data were then imported into Statistical Parametric Mapping (SPM) 12 (The Wellcome Trust College London, London, UK). Because the rs-fMRI data were acquired in an interleaved spatial order, slice-timing correction (with the second slice as reference since this slice was acquired first) was applied before head motion correction [42]. To improve the signal-to-noise ratio, the rs-fMRI images were spatially smoothed using a Gaussian kernel (full width at half maximum = 8 mm). Last, the rs-fMRI data were temporally filtered using FSL’s band-pass filter (0.01 to 0.1 Hz) [12] to remove possible respiratory and signal drift effects and to focus on the spontaneous low-frequency fluctuations.

The participant’s structural T1w images, including the masks of the CSF and WM, as well as the AAL atlas [37] were (affine) coregistered to the rs-fMRI data using FSL’s FLIRT [38], and an averaged time-series in each brain region as well as in the CSF and WM was calculated from the per-voxel time-series in each region.

Subsequently, for each pair of brain regions, a Pearson’s correlation coefficient was calculated using linear regression of the averaged time-series of each region, with the averaged time-series in the CSF and WM and the motion correction parameters as nuisance regressors in MATLAB Release 2016a (The Mathworks Inc., Natick, MA, USA). This resulted in a symmetric 94 × 94 correlation matrix, i.e., the participant’s functional connectome (FC). In the FC, each row and column represent a brain region and each element represents the temporal correlation between two regions. Last, the diagonal elements (self–self connections) and negative correlations, which are considered as not representing any meaningful connections, were set to zero [43], resulting in a correlation-weighted, undirected network.

### Appendix A.3. Hardware and Software

All structural and functional connectivity analyses were performed on a dedicated computer cluster containing four nodes, each with an Intel Xeon E3-1245v3 3.40 GHz 8-core processor, 32 GB of DDR4 RAM, and a 250 GB SSD. Processed data were stored on dedicated storage servers equipped with in total eight 10 TB SATA 6.0 Gb/s hard disks. To obtain a high level of data safety, the disks were configured in two RAID 6 arrays, thus providing protection against simultaneous failure of two disks and resulting in 20 TB of usable disk space. Each node was loaded with an image that contained the operating system, drivers, and dedicated software. The operating system was 64-bit Scientific Linux release 6.8 (Carbon) based on Linux kernel 2.6.32-642.11.1.el6.x86_64 and GNOME 2.28.2. The software included in the image analyses is listed in supplemental Table A1.

**Table A1:** Overview of neuro-imaging software used in the structural and functional connectivity processing pipelines.

Software Package	Version	Release Date	RRID
dcm2nii	2MAY2016	2 May 2016	SCR_014099
MATLAB including the Image Processing Toolbox	2016a	1 January 2016	SCR_001622
Brain Connectivity Toolbox	2017_01_15	15 January 2017	SCR_004841
ExploreDTI	4.8.6	23 February 2017	SCR_001643
FMRIB Software Library	5.0.10	25 April 2017	SCR_002823
Statistical Parametric Mapping	12 rev 6906	20 October 2016	SCR_007037

RRID: research resource identifier as reported by https://scicrunch.org.

### Appendix A.4. Processing Times and Size of Generated Data

Average processing times per participant in the structural and functional network connectivity pipelines were 1 h 33 m and 0 h 50 m, respectively. With the hard- and software used in this study, it took approximately 9 days to process the dMRI and rs-fMRI DICOM data of all participants. The amount of data generated per participant in the structural and functional network connectivity analyses were 847 MB and 744 MB, respectively, yielding a total of approximately 3.6 TB of generated data for the complete structural and functional connectivity analyses.

## Appendix B. Pearson’s Correlation Coefficients between dMRI and rs-fMRI Quality Metrics

**Table A2:** Pearson’s correlation coefficients between dMRI and rs-fMRI quality metrics.

		dMRI			Rs-fMRI		
		iSNR	Head Motion	Atlas Mismatch	iSNR	Head Motion	Atlas Mismatch
dMRI	iSNR	1	0.298 ***	0.114 ***	0.176 ***	0.248 ***	−0.052 ***
	Head motion		1	0.217 ***	0.247 ***	0.210 ***	−0.255 ***
	Atlas mismatch			1	0.112 ***	0.116 ***	−0.023
rs-fMRI	iSNR				1	0.522 ***	−0.133 ***
	Head motion					1	−0.114 ***
	Atlas mismatch						1

Abbreviations: iSNR: inverse signal-to-noise ratio. Significant at *** *p* < 0.001.

## Appendix C. Standardized Regression Coefficients and Goodness-of-Fit Parameters between the Connectivity Measures and the Image Quality Metrics

**Table A3:** (**A**) Standardized regression coefficients and goodness-of-fit parameters between the structural connectivity measures and the image quality metrics of the diffusion MRI. (**B)** Standardized regression coefficients and goodness-of-fit parameters between the functional connectivity measures and the image quality metrics of the functional MRI.

(A) Independent Variables	Overall SC		ν		γ		ε_global_	
	β	*p*-Value	β	*p*-Value	β	*p*-Value	β	*p*-Value
iSNR	−0.127	<0.001	−0.030	0.027	0.027	0.039	0.015	0.286
Head motion	−0.302	<0.001	−0.303	<0.001	0.372	<0.001	0.087	<0.001
Atlas mismatch	−0.015	0.253	−0.204	<0.001	0.105	<0.001	–0.169	<0.001
R^2^	0.132		0.167		0.173		0.30	
**(B) Independent** **Variables**	**Overall FC**		**ν**		**γ**		**ε_global_**	
	**β**	***p*** **-Value**	**β**	***p*** **-Value**	**β**	***p*** **-Value**	**β**	***p*** **-Value**
iSNR	−0.093	<0.001	−0.114	<0.001	0.105	<0.001	0.043	0.008
Head motion	0.053	0.001	−0.022	0.176	−0.028	0.087	0.127	<0.001
Atlas mismatch	0.011	0.445	0.020	0.163	0.011	0.451	−0.075	<0.001
R^2^	0.006		0.017		0.008		0.032	

Model: Connectivity measure = β_0_ + β_1_ * iSNR + β_2_ * Head motion + β_3_ * Atlas mismatch. Abbreviations: iSNR: inverse signal-to-noise ratio.

## Appendix D. Explanation for 0.1 mm of Head Motion being Equivalent to 18.3 Years of Aging

From the standard deviations for age and head motion in dMRI as reported in Table 1 and the standardized regression coefficients as reported in Table 2, we can calculate how many years of aging has the equivalent effect on overall structural connectivity as a given amount of millimeters of head motion.

From Table 2, we obtain the standardized regression coefficients for the complete model:

Overall structural connectivity = β_0_ − 0.074 * Age + 0.213 * Sex − 0.045 * Educational level + 0.109 * BMI + 0.003 * Diabetes status − 0.010 * History of CVD + 0.048 * WMH volume − 0.120 * iSNR − 0.249 * Head motion + 0.021 * Atlas mismatch

This can be interpreted as “for one SD years increase in age, the overall structural connectivity will decrease by 0.074 SD, and for one SD mm increase in head motion, the overall structural connectivity will decrease by 0.249 SD”.

From Table 1, we obtain the standard deviations for age and head motion, which are 8.7 years and 0.16 mm, respectively. Thus, for each 8.7 years of aging, overall structural connectivity decreases by 0.074 SD, and for each additional 0.16 mm of head motion, overall structural connectivity decreases by 0.249 SD.

To have a decrease of one SD in overall structural connectivity, we would need 117.6 (=8.7 years * 1SD/0.074SD) years of aging, or 0.643 (= 0.16 mm * 1SD/0.249SD) millimeters of head motion. And thus, 117.6 years of aging has the equivalent effect on overall structural connectivity 0.643 mm of head motion, which is the same as follows: 18.3 (117.6/6.43) years of aging has the equivalent effect on overall structural connectivity as 0.1 (=0.643/6.43) mm of head motion.

## Appendix E. Post Hoc Analyses with Cases of dMRI Motion > 1.0 mm Excluded

**Table A4:** Demographic and clinical characteristics of participants with of dMRI motion > 1.0 mm (n = 121).

Characteristic	
*Demographic*	
Age [mean (SD), years]	67.6 (6.7)
Sex [%] Male Female	89.3 10.7
Educational level [%] Low Medium High	44.2 21.7 34.2
*Clinical*	
BMI [mean (SD), kg/m^2^]	27.0 (3.7)
Diabetes status [%] No diabetes Prediabetes Type 2 diabetes Other type of diabetes	41.8 19.7 36.1 2.5
History of CVD [%] No Yes	75.6 24.4
Relative WMH volume [median (25–75th percentile), % of ICV]	0.083 (0.024–0.277)

Abbreviations: SD: standard deviation; BMI: body mass index; CVD: cardiovascular disease; WMH: white matter hyperintensities; ICV: intracranial volume.

**Table A5:** Standardized regression coefficients from linear regression model with structural connectivity measures as dependent variable and dMRI motion as independent variable, reported for all cases (original analyses, n = 5110) and for cases with motion ≤ 1.0 mm (n = 4989). All *p* < 0.001.

Structural Connectivity Measure	All Cases (Original Analyses)	Large Motion Cases Excluded
Overall structural connectivity	−0.343	−0.315
Average node degree (ν)	−0.356	−0.289
Normalized clustering coefficient (γ)	0.403	0.321
Normalized global efficiency (ε_global_)	0.054	0.068

**Table A6:** Standardized regression coefficients of the crude regression model between normalized clustering coefficient and the demographic/clinical variables (Model 1), and the same model with additional adjustment for the diffusion MR image quality metrics (Model 2). For significant regression coefficients, the percentage change is also calculated.

Independent Variables	All Cases (Original Analyses)			Large Motion Cases Excluded		
	Model 1	Model 2	∆ [%]	Model 1	Model 2	∆ [%]
Age	0.179 ***	0.059 ***	−67	0.156 ***	0.063 ***	−60
Sex	−0.207 ***	−0.096 ***	−54	−0.191 ***	−0.101 ***	−47
Educational level	−0.007	−0.007	-	−0.011	-0.011	-
BMI	−0.037 *	−0.054 ***	+46	−0.035 *	−0.055 ***	+57
Diabetes status	0.074 ***	0.046 **	−38	0.073 ***	0.052 ***	−29
History of CVD	0.021	0.014	-	0.014	0.010	-
WMH volume ^†^	0.093 ***	0.077 ***	−17	0.077 ***	0.075 ***	–3
iSNR	-	0.006	-	-	0.024	-
Head motion	-	0.310 ***	-	-	0.227 ***	-
Atlas mismatch	-	0.081 ***	-	-	0.085 ***	-
R^2^	0.121	0.199	+64	0.096	0.144	+50

Model (1) Normalized clustering coefficient = β0 + β1 * Age + β2 * Sex + β3 * Educational level + β4 * BMI + β5 * Diabetes status + β6 * History of CVD + β7 * WMH volume. Model (2) Normalized clustering coefficient = Model 1 + β8 * iSNR + β9 * Head motion + β10 * Atlas mismatch. Abbreviations: BMI: body mass index; CVD: cardiovascular disease; WMH: white matter hyperintensity volume; iSNR: inverse signal-to-noise ratio. ^†^ Log10-transformed. Significant at * *p* < 0.05; ** *p* < 0.01; *** *p* < 0.001.

**Table A7:** Standardized regression coefficients obtained from a linear regression model with forward selection between demographic/clinical variables and each of the quality metrics for the diffusion MRI.

Independent Variables	All Cases (Original Analyses)		Large Motion Cases Excluded	
	β	*p*–Value	β	*p*–Value
Age	0.368	<0.001	0.377	<0.001
Sex	−0.279	<0.001	−0.278	<0.001
Educational level	0.001	0.916	0.004	0.731
BMI	0.028	0.031	0.045	0.001
Diabetes status	0.095	<0.001	0.099	<0.001
History of CVD	0.020	0.093	0.015	0.212
WMH volume ^†^	0.048	<0.001	0.011	0.863
R^2^	0.297		0.295	

Abbreviations: BMI: body mass index; CVD: cardiovascular disease; WMH: white matter hyperintensity volume; iSNR: inverse signal-to-noise ratio. ^†^ Log10-transformed.
